# Discovery of the pyridylphenylureas as novel molluscicides against the invasive snail *Biomphalaria straminea*, intermediate host of *Schistosoma mansoni*

**DOI:** 10.1186/s13071-018-2868-7

**Published:** 2018-05-09

**Authors:** Weisi Wang, Qiang Mao, Junmin Yao, Weijia Yang, Qiming Zhang, Wencheng Lu, Zhuohui Deng, Liping Duan

**Affiliations:** 1 National Institute of Parasitic Diseases, Chinese Center for Disease Control and Prevention, WHO Collaborating Centre for Malaria, Schistosomiasis and Filariasis, Key Laboratory of Parasitology and Vector Biology of the Chinese Ministry of Health, Shanghai, 200025 China; 2 Guangdong Provincial Center for Disease Control and Prevention, WHO Collaborating Centre for Surveillance, Research and Training of Emerging Infectious Diseases, Guangzhou, 511430 Guangdong China; 3 College of Life and Environmental Sciences, Shanghai Normal University, Shanghai, 200234 China; 4 Institute of Microbiology, Chinese Academy of Sciences, Beijing, China

**Keywords:** *Biomphalaria straminea*, *Schistosoma mansoni*, Molluscicide, Pyridylphenylureas, Toxicity to fish

## Abstract

**Background:**

The snail *Biomphalaria straminea* is one of the intermediate hosts of *Schistosoma mansoni*. *Biomphalaria straminea* is also an invasive species, known for its strong capability on peripheral expansion, long-distance dispersal and colonization. Using molluscicides to control snail populations is an important strategy to interrupt schistosomiasis transmission and to prevent the spread of the invasive species. In this study, a series of pyridylphenylurea derivatives were synthesized as potential molluscicides. Their impact on adult snails and egg masses was evaluated. Acute toxicity to fish of the derivatives was also examined to assess their effect on non-target organisms. The preliminary mechanisms of action of the derivatives were studied by enzyme activity assays.

**Results:**

The representative compounds, 1-(4-chlorophenyl)-3-(pyridin-3-yl)urea (compound 8) and 1-(4-bromophenyl)-3-(pyridin-3-yl)urea (compound 9), exhibited strong molluscicidal activity against adult snails with LD_50_ values of 0.50 and 0.51 mg/l and potent inhibitory effects on snail egg hatchability with IC_50_ values of 0.05 and 0.09 mg/l. Notably, both compounds showed good target specificity with potent molluscicidal capability observed in snails, but very low toxicity to local fishes. Furthermore, the exposure of compounds 8 and 9 significantly elevated the enzyme activities of acid phosphatase and nitric oxide synthase of the snails, while no significant change was recorded in the activities of alkaline phosphatase, acetylcholine esterase and superoxide dismutase.

**Conclusion:**

The results suggested that compounds 8 and 9 of pyridylphenylurea derivatives could be developed as promising molluscicide candidates for snail control.

**Electronic supplementary material:**

The online version of this article (10.1186/s13071-018-2868-7) contains supplementary material, which is available to authorized users.

## Background

Schistosomiasis is a water-borne disease caused by the trematodes of the genus *Schistosoma*. It is the second most socio-economically devastating tropical disease after malaria [1]. Among the pathogenic schistosomes, *Schistosoma mansoni* is the most widespread species. It is found predominantly in Africa, South America, the Caribbean and the Middle East, and infects over 83 million people worldwide [2, 3]. The aquatic snails of the genus *Biomphalaria* act as intermediate hosts of *S. mansoni*. *Biomphalaria straminea* is an intermediate host species in the transmission of *S. mansoni*. Compared with its congeners, *B. straminea* is known for its strong capability on peripheral expansion, long-distance dispersal and colonization [4, 5]. A prehistoric distribution of *B. straminea* was found in Neotropical South America, mainly in Brazil [4]. In the last century, it has undergone peripheral range expansion to invade the Caribbean and other Neotropical countries [6]. Over the last decades, the distributional range of the snail has increased to outside the Neotropics [4]. *Biomphalaria straminea* was introduced to Hong Kong as an invasive species in 1973 and subsequently spread over the adjacent territories to Shenzhen City, southern China, in 1981 [7, 8]. By 2013, the snail had colonized a large area of Shenzhen City and overspread along the rivers to the surrounding areas, Dongguan City and Huizhou City, which geographically crosses the Zhujiang River Basin in southern China [9, 10]. The global distribution of human schistosomiasis coincides with the geographical distribution of the intermediate snail hosts [2]. Historically, China is a non-endemic area for *S. mansoni*; however, the aggressive colonization power of *B. straminea* raises concerns of both governmental and non-governmental organizations. In addition, due to the rapid development of international trade, flourishing tourism, frequent international personnel exchanges and labor export, a sharp increase in the numbers of imported *S. mansoni* cases in China has been observed since the 1990s [11, 12]. With such aggressive activity, close attention must be paid to the potential risk of the transmission of *S. mansoni* in mainland China.

As a part of an integrated schistosomiasis control programme, snail control strategies are considered an effective way to interrupt schistosomiasis transmission [13], and the application of molluscicides is the most widely used intervention strategy [14]. At present, efforts used to reduce snail populations primarily use the molluscicide niclosamide, which is highly potent against all developmental stages of the snail. However, niclosamide is highly toxic to fish and some amphibians [15, 16]. For *B. straminea*, a species of freshwater snail, using niclosamide to reduce its population is not the best choice because of its hazardous effect on non-target aquatic organisms. Alternative molluscicides with low toxicity are urgently needed.

Pyridine is a pharmacophoric structure commonly seen in the chemicals used for pest control and crop protection, such as neonicotinoids and ryanodine receptor activators [17–19]. Nicotinanilide, a pyridine molluscicide, is active against a broad spectrum of gastropods including *Biomphalaria glabrata*, *Oncomelania hupensis*, *Australorbis glabratus* and *Lymnaea luteola* [20–24]. A major advantage of nicotinanilide is its target specificity. It is not lethally toxic to fishes, tadpoles and frogs in field applications [20]. However, few studies have addressed the relationship between nicotinanilide and *B. straminea*. In our previous work, the molluscicidal potential of nicotinanilide against *B. straminea* has been evaluated, while only weak molluscicidal activity was revealed. In order to improve the potency, a urea group, a privileged structure in insecticides [25], was introduced in the chemical structure of nicotinanilide to replace the amide group. In the current study, a series of pyridylphenylurea derivatives were designed and synthesized and their molluscicidal and ovicidal activity against *B. straminea* adult snails and egg masses were evaluated. Toxicity of the derivatives to fish was also tested to assess their effects on non-target organisms. In addition, in order to understand the mechanisms of action, the impact of the compounds on the enzyme activities of five vital enzymes of *B. straminea*, alkaline phosphatase (ALP), acid phosphatase (ACP), acetylcholinesterase (AChE), nitric oxide synthase (NOS) and superoxide dismutase (SOD), was examined.

## Methods

### Chemistry

The synthesis and structural characterization data of pyridylphenylureas are described in Additional file 1.

### Snails and molluscicidal activity test

Healthy *B. straminea* snails were collected from the Guancang River in Dongguan City (114°5'41"E, 22°55'20"N), Guangdong Province, southern China. Adult specimens (shell diameter = 0.55 ± 0.10 cm, weight = 48.67 ± 10.02 mg) of *B. straminea* snails were raised in plastic tanks (25 × 15 × 10 cm) containing dechlorinated water (pH 7.40 ± 0.05) at 25 ± 1 °C with a 12:12 h photoperiod. The snails were fed with commercial golden fish food. The tanks were cleaned at least twice a week and water was drained and refilled as needed to maintain water quality. The snails then acclimatized to laboratory conditions for 3 weeks. In the test tanks, adult snails were immersed in an aqueous solution of the test compounds at each concentration (final concentrations: 0.25, 0.5, 1, 2, 5 and 10 mg/l, concentration of DMSO was less than 0.01%). For preliminary tests, 30 snails were used for each concentration of each compound; for the LC_50_ tests of compounds 8 and 9, 50 snails were used for each concentration. After 72 h exposure, the tanks were decanted and the snails were rinsed three times with dechlorinated water and offered golden fish food. Test snails were then left in water for another 48 h as a recovery period and examined to assess mortality. Niclosamide was used as a positive control and DMSO was used as a negative control. Snails were considered dead according to one or more of the following criteria: discoloration; contraction of the hemolymph; absence of muscle contraction; hemorrhage and deterioration of the body tissues [26, 27]. The 50/90% lethal concentration (LC_50_/LC_90_) values were calculated by probit analysis.

### Egg hatchability test

Egg hatchability tests were performed using the 6–7 days-old pre-hatched eggs of *B. straminea* [28, 29]*.* Ten egg masses (about 200 embryos in total) were collected and placed in a Petri dish and immersed in the aqueous solution of the test compounds at each concentration (final concentrations: 0.05, 0.1, 0.25, 0.5 and 1.0 mg/l). After 24 h exposure, the egg masses were checked every 4 days up to 28 days with a stereomicroscope. For each test, the number of hatched embryos was examined and recorded, and egg hatchability was calculated.

### Scanning electron microscopy (SEM)

Snails were treated with compounds 8 and 9 or niclosamide (positive control) at sub-lethal concentrations (LC_50_) for 24 h. DMSO-treated snails were used as a negative control. At the end of the exposure period the tentacle, cephalopodium and mantle of the snails were rapidly separated under a stereomicroscope. They were washed twice in PBS and fixed in 2.5% glutaraldehyde in 0.2 M PBS (pH 7.2) for 24 h. Subsequently, the specimens were washed in PBS and cold distilled water and dehydrated by sequential incubations in ethanol (50–100%). Dehydrated specimens were finally immersed in acetone and isoamyl acetate, and dried using a transitional medium of liquid carbon dioxide. They were then coated with platinum by an ion-sputtering apparatus and inspected on an FEI Inspect S scanning electron microscope.

### Acute lethal toxicity to fish test

Local fishes (cyprinoid carps, tilapias and grass carps) were used in the test with average weights of 1.26 ± 0.37 g, 0.99 ± 0.47 g and 0.83 ± 0.21 g, respectively. The average lengths were 4.79 ± 0.45 cm, 4.07 ± 0.64 cm and 4.66 ± 0.37 cm. New collected fish were maintained in dechlorinated water for at least one week to get acclimatized to laboratory conditions. The density of test fish was in the range of 10 fish in each aquarium (10 l) for each concentration (final concentrations: 5, 10 and 20 mg/l). Water temperature was maintained at 23 ± 1 °C throughout the experiment. Test fish were exposed to serial concentrations of compounds 8 and 9 for 72 h. Niclosamide was used as a positive control and DMSO was used as a negative control. The vitality of the fish was checked four times a day. Dead fish were removed as soon as their death was confirmed. At the end of the exposure interval, final mortality records of treated and control groups were made.

### Enzyme activity assay

*Biomphalaria straminea* snails were treated with compounds 8 and 9 at corresponding LC_50_ for 24 h. Thereafter, the surviving snails were collected for biochemical assays. Niclosamide (0.1 mg/l) was used as a positive control and DMSO was used as a negative control. Soft tissues of *B. straminea* snails were homogenized in PBS (pH 7.4) and centrifuged at 8000× *rpm* for 5 min at 4 °C. The supernatant was collected for assay. The enzyme activities of ALP, ACP, AChE, NOS and SOD of the snails were examined following the experimental procedures described in the technical bulletins of corresponding assay kits purchased from Nanjing Jiancheng Bioengineering Institute, Nanjing, China.

### Statistical analysis

Significant differences between control and treatment groups were tested by one-way ANOVA using SPSS19.0 software. The LC_50_ and LC_90_ values were calculated by probit analysis with 95% confidence limits and chi-square values using SPSS19.0.

## Results

### Molluscicidal activity against *B. straminea* adult snails

**Fig. 1:**
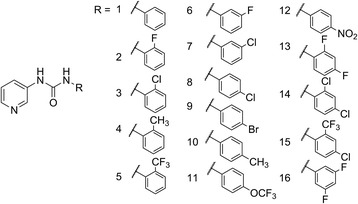
Chemical structures of pyridylphenylurea derivatives

A total of 16 pyridylphenylurea compounds (Fig. 1) were prepared and their molluscicidal potential against *B. straminea* adult snails was preliminarily screened at two concentrations (10 mg/l and 1 mg/l; Table 1). As we expected, all derivatives showed a mortality rate of 100% at the concentration of 10 mg/l. However, at the lower concentration of 1.0 mg/l, only two compounds with chlorine atom (compound 8) or bromine atom (compound 9) at the para-position of the phenyl group still exhibited strong molluscicidal capability, which were clearly superior to nicotinanilide. Compounds 8 and 9 demonstrated potent molluscicidal activity; their LC_50_ and LC_90_ values were comparable to those of niclosamide (Table 2). Complete mortality of all snails occurred within 10 to 14 h when exposed to niclosamide for compounds 8 and 9 it required 18 to 22 h after exposure.

**Table 1:** Molluscicidal activity of pyridylphenylureas against *B. straminea* adult snails

Compound	Mortality (%) (*n* = 30)	
	10 mg/l	1 mg/l
1	100	3.33
2	100	0
3	100	3.33
4	100	0
5	100	0
6	100	3.33
7	100	0
8	100	100
9	100	100
10	100	0
11	100	3.33
12	100	3.33
13	100	0
14	100	26.67
15	100	0
16	100	3.33
Nicotinanilide	100	24.0
Niclosamide	100	100
Control	0	0

**Table 2:** Molluscicidal activity and egg hatchability inhibitory activity of compounds 8 and 9

Compound	Adult snails				Egg hatchability			
	LC_50_ (mg/l)^a^	LC_90_ (mg/l)^a^	*χ* ^2b^	*P* ^b^	IC_50_ (mg/l)^a^	IC_90_ (mg/l)^a^	*χ* ^2b^	*P* ^b^
8	0.50 (0.44–0.57)	0.98 (0.82–1.25)	4.14	0.25	0.05 (0.038–0.056)	0.21 (0.19–0.25)	1.88	0.39
9	0.51 (0.46–0.57)	0.78 (0.69–0.97)	3.84	0.43	0.09 (0.081–0.111)	0.39 (0.35–0.44)	2.88	0.41
Niclosamide	0.114^c^/0.049^d^	0.212^c^/0.063^d^	**–**	**–**	0.01 (0.009–0.016)	0.06 (0.047–0.085)	1.18	0.56

^a^Values are given as means with 95% confidence intervals in parentheses

^b^Pearson chi-square goodness-of-fit test

^c^[31]

^d^[34]

### Inhibitory effect on snail egg hatchability

Based on the effectiveness on adult snails, compounds 8 and 9 were further evaluated for their impacts on egg hatchability under laboratory conditions. As highlighted in the experiment results (Table 2), egg hatchability rates of the treated groups were all significantly decreased compared to those of the negative control. The strength of the inhibitory effect of both compounds proved dose-dependent. After 24 h exposure, compounds 8 and 9 showed good and fast inhibitory ability on *B. straminea* egg hatchability, which both had comparability with niclosamide.

### Ultrastructural alterations

**Fig. 2:**
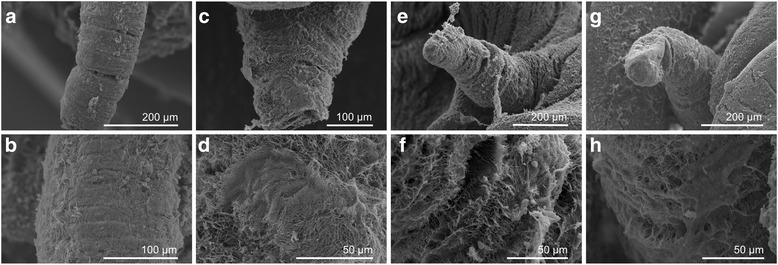
Scanning electron micrographs of *B. straminea* tentacles. Normal tentacles (**a**, **b**) with a smooth surface and fine cilia. Tentacles exposed to compounds 8 (**c**, **d**) and 9 (**e**, **f**) and niclosamide (**g**, **h**) showing a rough surface and disorderly distributed and extensively damaged cilia

**Fig. 3:**
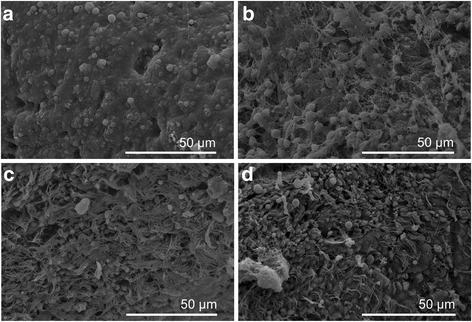
Scanning electron micrographs of *B. straminea* mantle. Normal mantle (**a**) showing smooth tegumental surface. Mantle exposed to compounds 8 (**b**) and 9 (**c**) and niclosamide (**d**) showing tortuosity, nipples, and erosion in the tegmental surface

**Fig. 4:**
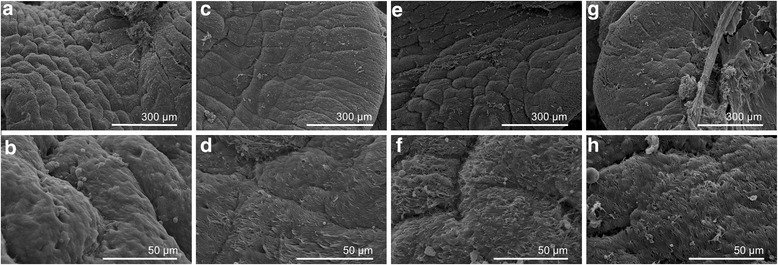
Scanning electron micrographs of *B. straminea* foot plantaris. The cilia of the normal foot plantaris (**a**, **b**) were smooth and regular. The cilia of the foot plantaris exposed to compounds 8 (**c**, **d**) and 9 (**e**, **f**) and niclosamide (**g**, **h**) became tangled and adherent

The potent molluscicidal ability of compounds 8 and 9 were further confirmed by the drug-induced severe ultrastructural alterations observed in the tentacles (Fig. 2), mantle (Fig. 3) and foot plantaris (Fig. 4) of *B. straminea* snails. SEM photomicrographs of the untreated soft body of *B. straminea* showed normal and intact tentacles; the tegumental surface of the mantle and foot plantaris were covered with fine and smooth cilia. Snails exposed to compounds 8 and 9 revealed marked ultrastructural destruction of the tentacles with exfoliation and exposure of sub-tegumental tissue. The tegumental surface of the mantle and foot plantaris was extensively damaged in the form of turgidity, blebbing, exfoliation, erosion and chap. Smooth and regular cilia were no longer apparent; instead, the cilia became tangled and adherent, and ultimately peeled off.

### Acute lethal fish toxicity

Local fishes were employed in the acute lethal fish toxicity test to ensure a practical application of the research. Results are summarized in Table 3. Generally, both compounds were well tolerated by local fishes at the exposure concentration of 5 mg/l, especially in the cyprinoid and grass carps. It is worth noting that, after 72 h exposure to compound 9, one of the test fish died at the highest concentration of 5 mg/l, which is 6 times higher than the absolute effective molluscicidal dose (LC_90_, 0.78 mg/l) and 13 times higher than the absolute effective egg hatchability inhibitory dose (IC_90_, 0.39 mg/l). Especially to cyprinoid and grass carps, the safe dose of compound 9 was lifted to 10 mg/l. During the entire exposure period, the test fish showed normal motor activity and food intake behavior at the tolerate exposure concentrations. No obvious toxic symptoms were observed including twitch bradykinesia, hypoxia, balance disorder or hemorrhage. In contrast, almost all the test fish died within 1 h after the exposure of niclosamide at the concentration of 0.2 mg/l.

**Table 3:** Acute lethal toxicity of compounds 8 and 9 on local fishes after 72 h exposure

Compound	Concentration (mg/l)	Mortality (%, *n* = 10)		
		Cyprinoid carp	Tilapia	Grass carp
8	5	0	50	0
	10	100	100	100
	20	100	100	100
9	5	0	0	0
	10	0	80	0
	20	100	100	100
Niclosamide	0.1	50	50	30
	0.2	100	100	80

### Effect on biochemical parameters of *B. straminea*

**Fig. 5:**
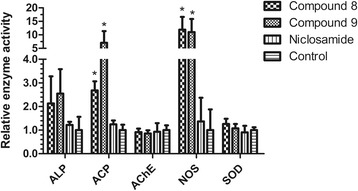
Changes of the enzyme activities of ALP, ACP, AChE, NOS and SOD of *B. straminea* snails exposed to compounds 8 and 9. Significant differences between control and treatment groups were tested by one-way ANOVA (ACP: *F*_(2, 15)_ = 6.37, *P = *0.019; NOS: *F*_(2, 15)_ = 12.04, *P < *0.001)

ALP, ACP, AChE, NOS and SOD are five vital enzymes involved in many biological processes of organisms. The activities of the five enzymes are often measured to evaluate the effects of molluscicides on target snails and determine possible mechanisms. As shown in Fig. 5, sub-lethal concentrations were enough to alter the biochemical parameters of the snail. The enzyme activities of ACP and NOS increased significantly after exposure to compound 9 when compared with those of the control group (ACP: *F*_(2, 15)_ = 6.37, *P=*0.019; NOS: *F*_(2, 15)_ = 12.04, *P < *0.001, Fig. 5). Although a 2-fold elevation occurred, there was no statistical difference in terms of ALP enzyme activity between control and compound 9-treated groups. No obvious change in the activities of AChE and SOD was observed. Compound 8 obtained similar results.

## Discussion

Niclosamide is a WHO recommended standard molluscicide used to combat intermediate host snails of schistosomes. However, niclosamide is far from an ideal molluscicide due to its various adverse effects. In Brazil, a traditional endemic area of *S. mansoni*, the application of niclosamide for the control of the *Biomphalaria* species has gradually decreased or even been discontinued in the last three decades mainly because of the rising global pressure for environmental protection and licensure difficulties to use environmentally hazardous substances in aquatic ecosystems [30]. Currently, the application of niclosamide is restricted to well-defined areas with high prevalences under close supervision [30]. There is an urgent need for safe and effective alternative molluscicides.

In this paper, we prepared a series of pyridylphenylureas with structural modifications focusing on the substituents of the phenyl group. Structure-activity relationship studies revealed that different substituents resulted in remarkably different influence on potency. Introducing halogens at the para-position of the phenyl group was beneficial for molluscicidal activity. Our study confirmed compounds 8 and 9 as effective molluscicides, killing *B. straminea* adult snails with LD_90_ values of 0.98 mg/l and 0.78 mg/l. It is worth noting that during the exposure to the two molluscicides no escape and/or avoidance behaviors of the snails were observed. In contrast, avoidance behaviors of the snails exposed to niclosamide and nicotinanilide have been described such as crawling out, water-leaving and aggregation at the water-air interface [31]. These behaviors hinder the action of molluscicides and eventually increase the snail’s chance of survival. Moreover, SEM photomicrographs illustrated the drug-induced morphological alterations. The two molluscicides resulted in ultrastructural destruction and extensive damage in both exposed (tentacle and foot plantaris) and shell-enclosed (mantle) tissues. Tentacles and foot plantaris are closely related to the vision, perception, muscle contraction and locomotor ability of the snail. Their damage strongly affects the food intake behavior, escape ability and hemolymph circulation of the snail. Mantle covers the visceral mass and protects the important organs, including heart, kidney, respiratory structures and albumin gland. Damaged mantle could no longer protect the internal organs from the toxicants, which leads to the death of the snail. Particularly, both molluscicides showed a pronounced impact on the cilia of the tegumental surface of the mantle and foot plantaris. The cilia became tangled and adherent instead of fine and smooth. In molluscs, cilia are strongly involved in both propulsion and the capture of food particles.

For snail control strategies, maintaining a low reproductive rate is of critical importance. Reducing egg hatchability is one effective measure of reproductive control. Our study demonstrates that the susceptibility of *B. straminea* to compounds 8 and 9 was independent of the snail developmental stage. Their inhibitory effects on egg hatchability were quite impressive with LC_90_ values of 0.21 mg/l and 0.39 mg/l. It was reported that the egg masses demonstrate a high degree of resistance to high molecular weight molluscicides, such as natural products and plant extracts, probably due to their poor penetrability of the gelatinous membrane [32, 33]. For compounds 8 and 9, small molecule synthetic molluscicides, it is much easier to penetrate the protective gelatinous membrane of the egg masses and act on the embryos.

As potential molluscicides used for snail control, we are interested in the toxic properties of pyridylphenylureas on snails, provided these are not hazardous to non-target aquatic organisms living in the same waters. Fishes are commonly used representative organisms in eco-toxicological studies. As expected, compounds 8 and 9 showed much higher therapeutic indices than niclosamide, which is another major achievement of the present study. At the effective molluscicidal concentrations, the two molluscicides were lethal to snails but not to local fishes. More attention will be focused on the impact of the two compounds on other aquatic animals and plants in our future studies.

ALP, ACP, AChE, NOS and SOD are five important enzymes implicated in several physiological and pathological processes and cover diverse biological functions. They are commonly used to study possible mechanisms of molluscicides [34–36]. The present results for compounds 8 and 9 indicate significant increases in the activities of ACP and NOS. The lack of an obvious effect on the activity of AChE clearly indicates that the main mechanism of action of the two molluscicides was not a cholinergic effect. In gastropods, ALP plays an important role in protein synthesis, secretion and shell formation [37, 38]. ACP, as a lysosomal enzyme, plays a critical role in catabolism, pathological necrosis and phagocytosis [37, 38]. Nitric oxide (NO), produced by the enzyme NOS, is a messenger molecule that displays important functions in the physiological process [39]. NOS is a highly conserved enzyme, which has been identified in snails, including *Biomphalaria glabrata*, *Lymnaea stagnalis* and *Helix pomatia* [40, 41]. It is clear from the present results that compounds 8 and 9 at sub-lethal concentrations disturb certain enzymes that are necessary for the normal metabolism and physiological activity of the snail which eventually lead to death.

## Conclusions

In this study, a series of pyridylphenylurea derivatives were prepared as potential molluscicides against the invasive snail species *B. straminea*. Among them, compounds 8 and 9 showed strong molluscicidal ability against adult snails and potent inhibitory effects on snail egg hatchability. It is noteworthy that the two compounds exhibited much lower toxicity to fish and much higher therapeutic indices than niclosamide and could be developed as promising molluscicide candidates. Field trials and further studies on the molecular mechanism of the two molluscicides are in progress.

## Additional file

Additional file 1: The synthesis and structural characterization data of pyridylphenylureas. (DOCX 157 kb)
