# Supplementary material for: Discovery of the pyridylphenylureas as novel molluscicides against the invasive snail Biomphalaria straminea, intermediate host of Schistosoma mansoni
Source: Parasit Vectors. 2018 May 9;11:291. doi: 10.1186/s13071-018-2868-7 (PMC5944108; doi:10.1186/s13071-018-2868-7)
Supplement: Supplementary file 1 — The synthesis and structural characterization data of pyridylphenylureas. (DOCX 157 kb) [file 13071_2018_2868_MOESM1_ESM.docx]

**Additional file 1**

**1. Chemistry**

Reagents and solvents were purchased from Sigma-Aldrich and were used without further purification. Melting points were measured with a B-540 Büchi apparatus and were uncorrected. ^1^H-NMR and ^13^C-NMR spectra were recorded on a Bruker AM-400 spectrometer (400 MHz). Chemical shifts are given in ppm (δ) relative to TMS as internal standard, and signals are used by the following abbreviations: s, singlet; d, doublet; t, triplet; m, multiplet, etc. High resolution mass spectrums (HRMS) were recorded on a Thermo Q Exactive Orbitrap LC-MS/MS. Thin layer chromatography (TLC) was carried out using plate silica gel F254 Merck. All yields are not optimized and generally represent the result of a single experiment.

**2. General procedures for the synthesis of pyridylphenylureas (Figure S1)**

To a solution of pyridin-3-amine (10 mmol) in CH_3_CN (40 mL), corresponding phenyl isocyanates (12 mmol) was added. After heating to 35 °C, triethylamine (1 mL) was added dropwise. The reaction mixture was heated to reflux for 6h. The progress of the reaction was monitored by TLC. After cooling to room temperature, the obtained precipitates were filtrated and washed with hot water (70 °C) three times, and then recrystallized from aqueous ethanol to afford target compounds **1-16**.

**Figure S1.** Synthetic routes of pyridylphenylureas

***1-Phenyl-3-(pyridin-3-yl)urea (1).*** Yield: 40%, white solid, mp 154.6-155.3 °C. ^1^H NMR (400 MHz, DMSO) δ 9.24 (m, 2H, NH and Ar-H), 8.48 (d, *J* = 2.8 Hz, 1H, Ar-H), 8.17 (dd, *J* = 8.8, 2.8 Hz, 1H, Ar-H), 7.49 (dd, *J* =5.2, 2.0 Hz, 2H, Ar-H), 7.44 (d, *J* = 8.4 Hz, 1H, Ar-H), 7.35 (d, *J* = 8.8 Hz, 2H, Ar-H), 7.27 (m, 1H, Ar-H). ^13^C NMR (100 MHz, DMSO) δ 152.62, 139.48, 139.44, 137.15, 133.47, 132.05, 129.30, 126.95, 123.06, 119.16. HRMS-ESI *m*/*z* [*M*+H]^+^ calcd for C_13_H_13_N_3_O:213.0902, found: 214.0973.

***1-(2-Fluorophenyl)-3-(pyridin-3-yl)urea*** ***(2).*** Yield: 87%, white solid, mp 183.4-185.0 °C. ^1^H NMR (400 MHz, DMSO) δ 8.66 (d, *J* = 2.5 Hz, 1H, Ar-H), 8.26 (dd, *J* = 4.7, 1.4 Hz, 1H, Ar-H), 8.18 (td, *J* = 8.2, 1.6 Hz, 1H, Ar-H), 8.02 (ddd, *J* = 8.3, 2.6, 1.5 Hz, 1H, Ar-H), 7.38 (dd, *J* = 8.3, 4.7 Hz, 1H, Ar-H), 7.30 (ddd, *J* = 11.6, 8.2, 1.4 Hz, 1H, Ar-H), 7.21 (t, *J* = 7.3 Hz, 1H, Ar-H), 7.11-7.06 (m, 1H, Ar-H). ^13^C NMR (100 MHz, DMSO) δ (153.36, 150.96), 152.27, 143.03, 139.87, 136.19, (127.29, 127.19), 124.99, (124.53, 124.49), 123.66, (122.85, 122.78), 120.84, (115.11, 114.92). HRMS-ESI *m*/*z* [*M*+H]^+^ calcd for C_12_H_10_FN_3_O: 231.0808, found: 232.0871.

***1-(2-Chlorophenyl)-3-(pyridin-3-yl)urea*** ***(3).*** Yield: 82%, pinkish solid, mp 185.6-186.6 °C. ^1^H NMR (400 MHz, DMSO) δ 9.01 (br, 1H, NH), 8.61 (d, *J* = 2.5 Hz, 1H, Ar-H), 8.21 (dd, *J* = 4.7, 1.4 Hz, 1H, Ar-H), 8.14 (dd, *J* = 8.3, 1.5 Hz, 1H, Ar-H), 7.97 (ddd, *J* = 8.3, 2.6, 1.5 Hz, 1H, Ar-H), 7.47 (dd, *J* = 8.0, 1.4 Hz, 1H, Ar-H), 7.35-7.29 (m, 2H, Ar-H), 7.06 (td, *J* = 7.7, 1.5 Hz, 1H, Ar-H). ^13^C NMR (100 MHz, DMSO) δ 152.73, 143.66, 140.47, 136.62, 135.69, 129.06, 128.12, 126.79, 125.63, 124.18, 123.51, 122.92. HRMS-ESI *m*/*z* [*M*+H]^+^ calcd for C_12_H_10_ClN_3_O: 247.0512, found: 248.0576.

***1-(Pyridin-3-yl)-3-(o-tolyl)urea (4).*** Yield: 89%, white solid, mp 160.2-160.4 °C. ^1^H NMR (400 MHz, DMSO) δ 9.22 (br, 1H, NH), 8.61 (d, *J* = 2.5 Hz, 1H, Ar-H), 8.18 (dd, *J* = 4.7, 1.4 Hz, 1H, Ar-H), 7.97 (ddd, *J* = 8.3, 2.6, 1.5 Hz, 1H, Ar-H), 7.80 (d, *J* = 7.3 Hz, 1H, Ar-H), 7.32 (dd, *J* = 8.3, 4.6 Hz, 1H, Ar-H), 7.20-7.14 (m, 2H, Ar-H), 7.00-6.95 (m, 1H, Ar-H), 2.25 (s, 3H, CH_3_). ^13^C NMR (100 MHz, DMSO) δ 153.22, 143.16, 140.28, 137.59, 137.12, 130.69, 128.46, 126.64, 125.32, 124.10, 123.50, 121.90, 18.34. HRMS-ESI *m*/*z* [*M*+H]^+^ calcd for C_13_H_13_N_3_O: 227.1059, found: 228.1122.

***1-(Pyridin-3-yl)-3-(2-(trifluoromethyl)phenyl)urea (5).*** Yield: 90%, white solid, mp 132.8-134.8 °C. ^1^H NMR (400 MHz, DMSO) δ 9.53 (s, 1H, NH), 8.61 (d, *J* = 2.5 Hz, 1H, Ar-H), 8.22-8.21 (m, 2H, Ar-H and NH), 7.98-7.93 (m, 2H, Ar-H), 7.71-7.64 (m, 2H, Ar-H), 7.35-7.29 (m, 2H, Ar-H). ^13^C NMR (100 MHz, DMSO) δ 152.57, 143.13, 139.90, 136.19, 135.97, 132.93, 125.95, 125.08, 124.05, 123.67, 120.37, 120.09. HRMS-ESI *m*/*z* [*M*+H]^+^ calcd for C_13_H_10_F_3_N_3_O: 281.0776, found: 282.0838.

***1-(3-Fluorophenyl)-3-(pyridin-3-yl)urea (6).*** Yield: 86%, white solid, mp 180.3-181.4 °C. ^1^H NMR (400 MHz, DMSO) δ 9.04 (br, 2H, NH), 8.61 (d, *J* = 2.5 Hz, 1H, Ar-H), 8.21 (dd, *J* = 4.7, 1.4 Hz, 1H, Ar-H), 7.94 (ddd, *J* = 8.3, 2.6, 1.5 Hz, 1H, Ar-H), 7.49 (dt, *J* = 12.0, 2.3 Hz, 1H, Ar-H), 7.35-7.29 (m, 2H, Ar-H), 7.15 (dd, *J* = 8.2, 1.2 Hz, 1H, Ar-H), 6.81 (td, *J* = 8.3, 2.3 Hz, 1H, Ar-H). ^13^C NMR (100 MHz, DMSO) δ (163.54, 161.14), 152.43, 143.07, (141.36, 141.25), 140.20, 136.13, (130.38, 130.28), 125.34, 123.59, 114.08, (108.48, 108.27), (105.14, 104.87). HRMS-ESI *m*/*z* [*M*+H]^+^ calcd for C_12_H_10_FN_3_O: 231.0808, found: 232.0872.

***1-(3-Chlorophenyl)-3-(pyridin-3-yl)urea*** ***(7).*** Yield: 88%, pinkish solid, mp 178.0-178.7 °C. ^1^H NMR (400 MHz, DMSO) δ 9.32 (br, 1H, NH), 8.63 (d, *J* = 1.9 Hz, 1H, Ar-H), 8.19 (d, *J* = 4.5 Hz, 1H, Ar-H), 7.96-7.93 (m, 1H, Ar-H), 7.73 (s, 1H, Ar-H), 7.32-7.26 (m, 3H, Ar-H), 7.03-7.01 (m, 1H, Ar-H). ^13^C NMR (100 MHz, DMSO) δ 153.14, 143.38, 141.77, 140.71, 136.90, 133.62, 130.81, 125.76, 124.02, 121.97, 118.15, 117.24. HRMS-ESI *m*/*z* [*M*+H]^+^ calcd for C_12_H_9_ClN_3_O: 247.0512, found: 248.0577.

***1-(4-Chlorophenyl)-3-(pyridin-3-yl)urea (8).*** Yield: 92%, white solid, mp 217.0-217.8 °C. ^1^H NMR (400 MHz, DMSO) δ 9.19 (br, 1H, NH), 8.62 (d, *J* = 2.5 Hz, 1H, Ar-H), 8.19 (d, *J* = 4.6 Hz, 1H, Ar-H), 7.94 (ddd, *J* = 8.3, 2.5, 1.5 Hz, 1H, Ar-H), 7.51 (d, *J* = 8.8 Hz, 2H, Ar-H), 7.34-7.29 (m, 3H, Ar-H). ^13^C NMR (100 MHz, DMSO) δ 152.60, 142.84, 140.13, 138.61, 136.41, 128.57, 125.44, 125.18, 123.53, 119.84. HRMS-ESI *m*/*z* [*M*+H]^+^ calcd for C_12_H_10_ClN_3_O: 247.0512, found: 248.0577.

***1-(4-Bromophenyl)-3-(pyridin-3-yl)urea (9).*** Yield: 86%, white solid, mp 228.8-230.3 °C. ^1^H NMR (400 MHz, DMSO) δ 9.05 (br, 1H, NH), 8.61 (d, *J* = 2.4 Hz, 1H, Ar-H), 8.19 (dd, *J* = 4.6, 1.3 Hz, 1H, Ar-H), 7.93 (ddd, *J* = 8.3, 2.5, 1.5 Hz, 1H, Ar-H), 7.48-7.43 (m, 4H, Ar-H), 7.32 (dd, *J* = 8.3, 4.7 Hz, 1H, Ar-H). ^13^C NMR (100 MHz, DMSO) δ 152.99, 143.41, 140.66, 139.43, 136.79, 131.97, 125.73, 124.02, 120.77, 120.70, 113.94. HRMS-ESI *m*/*z* [*M*+H]^+^ calcd for C_12_H_10_BrN_3_O: 291.0007, found: 292.0070.

***1-(Pyridin-3-yl)-3-(p-tolyl)urea (10).*** Yield: 84%, white solid, mp 182.4-184.1 °C. ^1^H NMR (400 MHz, DMSO) δ 9.06 (br, 1H, NH), 8.61 (d, *J* = 2.4 Hz, 1H, Ar-H), 8.17 (dd, *J* = 4.6, 1.3 Hz, 1H, Ar-H), 7.94 (ddd, *J* = 8.3, 2.6, 1.5 Hz, 1H, Ar-H), 7.36 (d, *J* = 8.4 Hz, 2H, Ar-H), 7.30 (dd, *J* = 8.3, 4.7 Hz, 1H, Ar-H), 7.09 (d, *J* = 8.3 Hz, 2H, Ar-H), 2.25 (s, 3H, CH_3_). ^13^C NMR (100 MHz, DMSO) δ 152.97, 143.13, 140.43, 137.39, 137.06, 131.32, 129.64, 125.43, 124.03, 118.89, 20.82. HRMS-ESI *m*/*z* [*M*+H]^+^ calcd for C_13_H_13_N_3_O: 227.1059, found: 228.1120.

***1-(Pyridin-3-yl)-3-(4-(trifluoromethoxy)phenyl)urea (11).*** Yield: 91%, white solid, mp 178.3-178.7 °C. ^1^H NMR (400 MHz, DMSO) δ 9.08 (br, 2H, NH), 8.62 (d, *J* = 2.5 Hz, 1H, Ar-H), 8.20 (dd, *J* = 4.7, 1.4 Hz, 1H, Ar-H), 7.94 (ddd, *J* = 8.3, 2.5, 1.5 Hz, 1H, Ar-H), 7.59-7.55 (m, 2H, Ar-H), 7.34-7.29 (m, 3H, Ar-H). ^13^C NMR (100 MHz, DMSO) δ 152.54, 142.97, 142.72, 140.17, 138.76, 136.26, 125.26, 123.57, 121.70, 119.57. HRMS-ESI *m*/*z* [*M*+H]^+^ calcd for C_13_H_10_F_3_N_3_O_2_: 297.0725, found: 298.0748.

***1-(4-Nitrophenyl)-3-(pyridin-3-yl)urea (12).*** Yield: 90%, yellow solid, mp 224.4-226.1 °C. ^1^H NMR (400 MHz, DMSO) δ 8.68 (d, *J* = 2.5 Hz, 1H, Ar-H), 8.32 (s, 1H, NH), 8.18 (dd, *J* = 4.7, 1.4 Hz, 1H, Ar-H), 8.16-8.12 (m, 2H, Ar-H), 7.98 (ddd, *J* = 8.4, 2.5, 1.5 Hz, 1H, Ar-H), 7.73-7.69 (m, 2H, Ar-H), 7.30 (dd, *J* = 8.3, 4.6 Hz, 1H, Ar-H). ^13^C NMR (100 MHz, DMSO) δ 152.96, 143.26, 142.72, 140.81, 137.22, 134.33, 125.72, 125.55, 123.98, 118.25. HRMS-ESI *m*/*z* [*M*+H]^+^ calcd for C_12_H_10_N_4_O_3_: 258.0753, found: 259.0816.

***1-(2,4-Difluorophenyl)-3-(pyridin-3-yl)urea (13).*** Yield: 80%, white solid, mp 191.5-193.8 °C. ^1^H NMR (400 MHz, DMSO) δ 8.60 (d, *J* = 2.5 Hz, 1H, Ar-H), 8.20 (dd, *J* = 4.7, 1.4 Hz, 1H, Ar-H), 8.04 (td, *J* = 9.2, 6.2 Hz, 1H, Ar-H), 7.95 (ddd, *J* = 8.3, 2.6, 1.5 Hz, 1H, Ar-H), 7.34-7.29 (m, 2H, Ar-H), 7.08-7.03 (m, 1H, Ar-H). ^13^C NMR (100 MHz, DMSO) δ (158.29, 155.89), (153.71, 151.28), 152.41, 143.02, 139.90, 136.18, 125.01, (123.84, 123.73), 123.62, (122.47, 122.38), (111.16, 110.94), (104.06, 103.79). HRMS-ESI *m*/*z* [*M*+H]^+^ calcd for C_12_H_9_F_2_N_3_O: 249.0714, found: 250.0776.

***1-(2,4-Dichlorophenyl)-3-(pyridin-3-yl)urea*** ***(14).*** Yield: 85%, white solid, mp 186.8-189.0 °C. ^1^H NMR (400 MHz, DMSO) δ 8.61 (d, *J* = 2.4 Hz, 1H, Ar-H), 8.22 (dd, *J* = 4.7, 1.4 Hz, 1H, Ar-H), 8.18 (d, *J* = 9.0 Hz, 1H, Ar-H), 7.96 (ddd, *J* = 8.3, 2.5, 1.5 Hz, 1H, Ar-H), 7.63 (d, *J* = 2.4 Hz, 1H, Ar-H), 7.39 (dd, *J* = 9.0, 2.5 Hz, 1H, Ar-H), 7.34 (dd, *J* = 8.3, 4.7 Hz, 1H, Ar-H). ^13^C NMR (100 MHz, DMSO) δ 153.05, 143.34, 140.61, 139.07, 138.84, 136.87, 129.06, 125.95, 125.65, 124.02, 121.33, 120.32. HRMS-ESI *m*/*z* [*M*+H]^+^ calcd for C_12_H_9_ Cl_2_N_3_O: 281.0123, found: 282.0188.

***1-(4-Chloro-2-(trifluoromethyl)phenyl)-3-(pyridin-3-yl)urea*** ***(15).*** Yield: 81%, white solid, mp 160.0-162.3 °C. ^1^H NMR (400 MHz, DMSO) δ 8.62 (d, *J* = 2.5 Hz, 1H, Ar-H), 8.18 (d, *J* = 4.6 Hz, 1H, Ar-H), 8.00-7.96 (m, 2H, Ar-H), 7.70-7.67 (m, 2H, Ar-H), 7.30 (dd, *J* = 8.3, 4.7 Hz, 1H, Ar-H). ^13^C NMR (100 MHz, DMSO) δ 153.07, 143.64, 140.40, 136.68, 136.49, 133.45, 128.79, 126.46, 126.43, 125.58, 124.56, 124.18. HRMS-ESI *m*/*z* [*M*+H]^+^ calcd for C_13_H_9_ClF_3_N_3_O: 315.0386, found: 316.0449.

***1-(3,5-Difluorophenyl)-3-(pyridin-3-yl)urea*** ***(16).*** Yield: 88%, pinkish solid, mp 203.0-205.3 °C. ^1^H NMR (400 MHz, DMSO) δ 8.62 (d, *J* = 2.5 Hz, 1H, Ar-H), 8.19 (dd, *J* = 4.7, 1.3 Hz, 1H, Ar-H), 7.94 (ddd, *J* = 8.3, 2.5, 1.5 Hz, 1H, Ar-H), 7.30 (dd, *J* = 8.3, 4.7 Hz, 1H, Ar-H), 7.22 (dd, *J* = 10.0, 2.2 Hz, 2H, Ar-H), 6.77 (tt, *J* = 9.4, 2.3 Hz, 1H, Ar-H). ^13^C NMR (100 MHz, DMSO) δ (164.30, 164.15), (161.90, 161.74), 153.36, 143.32, 140.81, 137.04, 125.81, 123.95, (101.58, 101.29), (97.19, 96.93, 96.66). HRMS-ESI *m*/*z* [*M*+H]^+^ calcd for C_12_H_9_F_2_N_3_O: 249.0714, found: 250.0777.
